# Delivery of Porphyrins Through Self-Assembling Peptide Hydrogels for Accelerated Healing of Experimental Skin Defects In Vivo

**DOI:** 10.7759/cureus.39120

**Published:** 2023-05-17

**Authors:** Ismene A Dontas, Pavlos Lelovas, Sofia Parara, Antonios Galanos, Georgios Agrogiannis, Dimitris Goutas, Georgios Charalambidis, Vasilis Nikolaou, Georgios Landrou, Chrysoula Kokotidou, Chrysanthi-Pinelopi Apostolidou, Anna Mitraki, Athanassios G Coutsolelos

**Affiliations:** 1 Veterinary Medicine, Laboratory for Research of the Musculoskeletal System, School of Medicine, National and Kapodistrian University of Athens, Athens, GRC; 2 Plastic Surgery, Laboratory for Research of the Musculoskeletal System, School of Medicine, National and Kapodistrian University of Athens, Athens, GRC; 3 Epidemiology and Public Health, Laboratory for Research of the Musculoskeletal System, School of Medicine, National and Kapodistrian University of Athens, Athens, GRC; 4 First Department of Pathology, School of Medicine, National and Kapodistrian University of Athens, Athens, GRC; 5 Laboratory of Bioinorganic Chemistry, Department of Chemistry, University of Crete, Voutes University Campus, Heraklion, GRC; 6 Department of Materials Science and Technology, University of Crete, Voutes University Campus, Heraklion, GRC; 7 Institute of Electronic Structure and Laser, Foundation for Research and Technology - Hellas, Heraklion, GRC

**Keywords:** wound healing, skin defect, self-assembling peptide, porphyrin, planimetry, hydrogel, histology

## Abstract

Introduction: The care and healing of skin defects resulting from different causes has been the object of research to achieve rapid and complete skin regeneration. Hydrogels have been used for their ability to maintain hydration during wound healing, absorb wound exudate, and cover the underlying tissue without adherence while being transparent. In this study, we evaluated the efficacy of a hydrogel (H) with encapsulated porphyrin (H+P) on a rat model of surgically-induced skin defects.

Methods: Four round 6 mm diameter skin defects were performed under general anesthesia on the dorsal area of 24 three-month-old "Young" and 24 twelve-month-old "Mature" male rats. Each age group was separated into the Control, H, and H+P groups, n=8 each, where no therapy, H, or H+P was respectively applied daily for 20 days. Digital photographs and skin biopsies were taken on the third, seventh, 10th, and 20th postoperative days and evaluated by planimetry, histology, and immunohistochemistry.

Results: Planimetry results demonstrated significantly decreased perimeter, diameter, and area measurements (p<0.005) of group H+P compared to Control and H groups on days 10 and 20 in the young rats, while in the mature rats, the significant differences were evident earlier (perimeter third day p<0.05; diameter and area seventh day p<0.05 and p<0.005, respectively vs. H). Granulation and scar tissue formation were also reduced in the H+P groups although they were not statistically significant.

Conclusions: The application of H+P on the skin defects benefited the healing process in both young and mature animal groups, as evidenced by the statistically significant findings of planimetry. The beneficial healing process was more pronounced in the mature animals, both in the level of statistical significance as well as regarding time (evident already on the third day of healing), probably due to porphyrin assisting the reduced healing rate, which is observed in organisms of advanced age.

## Introduction

The care and healing of skin defects, which may occur from trauma, resection of neoplasia, or burn wounds, have continuously been the object of research to prevent infection and accelerate the regeneration of these defects [1,2]. Since decades ago, several options have been available for skin wound management [3]; however, during the healing process, problems may often occur in clinical practice, as there is no specific drug, dressing, or therapeutic regimen that can be applied to all cases. It is not uncommon that after some time of treatment with an effective agent, wound healing begins to delay, and the clinician has to decide whether to continue or change to a new one [3].

Management of extensive skin defects has been shown to be achieved through surgical procedures and modern skin replacement strategies which can produce successful functional and cosmetic results, superior to those achieved through conservative measures. These include and are not limited to autologous full-thickness or split-thickness skin grafts, allografts, and xenografts [2]. In addition to these surgical methods of skin defect closure, the application of substances such as hydrocolloid dressings or hydrogels that promote wound healing has been the object of investigation [4]. Amongst their attractive properties are their biocompatibility, their ability to maintain an ideal hydration environment for wound healing, absorb wound exudate, cover the sensitive underlying tissue without adherence, provide gaseous permeability of traditional gauze dressings while being transparent, and allow observation of the healing process [4-7].

With this breadth of potential, various active hydrogel biomaterials are being synthesized with the objective to provide a beneficial environment to promote wound healing [6,8,9]. Carbohydrate-based hydrogels based on chitosan, hyaluronic acid, etc. are most frequently used for wound healing; however, self-assembling peptide hydrogels are recently gaining increasing interest as biomaterials suitable for tissue regeneration and wound healing [4,10]. Peptide-based hydrogels meet the basic requirements regarding the bioavailability and bioactivity of drugs in the pharmaceutical industry. In addition, it is possible to encapsulate within them drugs or chromophores that possess antibacterial and other pharmaceutical properties. The dipeptide phenylalanine-phenylalanine (FF) is a minimal self-assembling building block that forms hydrogels when protected at the N-terminal end with the protecting group Fmoc (9-fluorenyl-methoxy-carbonyl) that acts as a potent hydrogelator. The Fmoc-FF hydrogels were found to support cell adhesion and proliferation; they were also assessed for their ability to encapsulate and release small molecules in a controlled manner [11]. They were therefore deemed suitable carriers for biomedical applications such as controlled drug delivery [12].

The aim of the present study was to evaluate the effectiveness and safety of the application of Fmoc-FF hydrogels with encapsulated porphyrin, on the healing of skin defects in a rat model. Porphyrins were selected due to their unique properties, namely their photo-stability, strong absorption, and their ability to generate reactive oxygen species [13-15]. Indeed, porphyrinoids (metallated or free-base) have been utilized as photosensitizers to eradicate different families of microorganisms [16,17]. Diphenylalanine dipeptide (Fmoc-FF), as mentioned above, can form hydrogels with excellent stability and is widely studied due to its chemical simplicity and versatility [18].

In order to evaluate the healing properties of this combination, we used the rat model of a full-thickness dorsal skin round wound of 6 mm diameter. Wound healing has been studied extensively in rats and their epidermis, basement membrane, and dermis are comparable to that of humans [19]. Although rats present a subcutaneous *panniculus carnosus* muscle, which contributes to their wound healing by contraction and collagen formation, and which is absent in humans, this limitation has been overlooked in skin healing studies due to their multiple advantages [19]. Taking this difference under consideration, in our study we evaluated the healing process of the round wound's diameter, perimeter, and area as the ratio of the final value of each parameter to the initial value of the deficit by a planimetry software system [20]. Additionally, this evaluation was conducted on both young and mature rats, in order to observe potential differences due to aging.

## Materials and methods

Laboratory animals

The protocol was evaluated by the Protocol Evaluation Committee of the Laboratory for Research of the Musculoskeletal System, School of Medicine, National & Kapodistrian University of Athens, Greece, and licensed (6651/07-12-2018) by the General Directorate of Veterinary Services according to national legislation (Presidential Decree 56/2013, in compliance with the European Directive 2010/63/EU). Following a sample size estimation, performed using G*Power 3.1.9.2 program (Heinrich-Heine-Universität Düsseldorf, Düsseldorf, Germany), a total of 48 male Wistar rats were purchased from the breeding establishment of the Hellenic Pasteur Institute, Athens, Greece, at the age of three months. It was also determined that eight rats per group were required for an 80% probability of demonstrating a difference between groups of >20% between the Control and the groups receiving the active substance on the seventh postoperative day with a significance of α=0.016 (two-tailed test with Bonferroni correction).

Twenty-four animals were allocated to the “Young” rat group (three months of age) and 24 to the “Mature” rat group (12 months of age). Before surgery, the animals were housed three per cage in transparent polycarbonate cages (open top cages, 45×30×20 cm; Tecniplast SPA, Buguggiate, Varese, Italy) and maintained under standard laboratory conditions (temperature of 19 to 22°C; relative humidity of 55-65%, 15 air changes per hour, light/dark cycle at 06:00/18:00 hours). After surgery, they were housed in double-decker individually ventilated cages for rats (Tecniplast), three per cage, in order to diminish potential friction or trauma to their dorsal area from the food hopper of the standard open-top cages previously used.

Surgery, creation of skin defects, and application of substances

According to the protocol, all animals were anesthetized under general anesthesia (intramuscular injection of 50 mg/kg ketamine hydrochloride and 0.25 mg/kg dexmedetomidine), placed in ventral recumbency, their neck area hair clipped, and a subcutaneous injection of enrofloxacin (10 mg/kg) and butorphanol (2 mg/kg) were administered in the lumbar area (avoiding the dorsal neck area where the defects would be performed).

**Figure 1:**
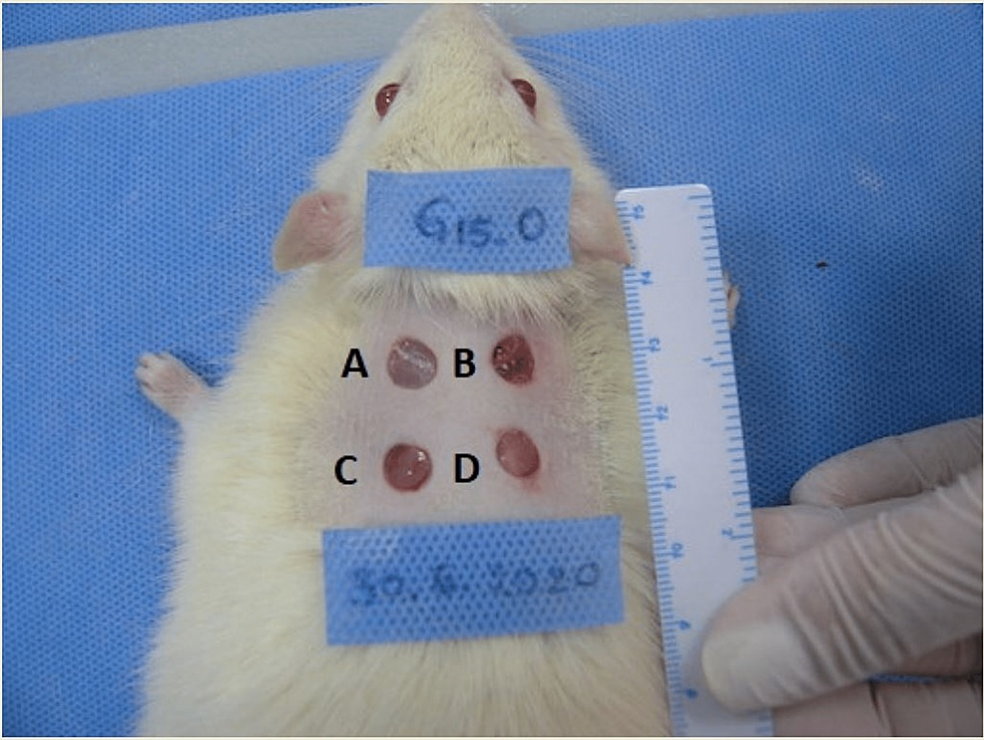
Creation of the four skin defects at baseline (day 0) by a round skin punch of 6 mm diameter.

In both young and mature animals, four skin defects (A, B, C, and D) were created with a round skin punch (Kai Medical biopsy punch; Kai Industries Co. Ltd, Tokyo, Japan) with a diameter of 6 mm and depth of total thickness in the area 1.5-3 cm caudally from the occipital bone protuberance along the spine, so that they would not be accessible by the animals to lick (Figure 1). It was necessary to have one defect for each examination day planned (third, seventh, 10th, and 20th postoperative days) in order to monitor the defects' healing throughout the 20-day healing period, so that the respective defect photograph for planimetry (with its perimeter, diameter, and area) and especially the punch biopsy for the histological evaluation of the healing process, would include a whole defect.

The 24 animals of each age category were allocated eight to each of the following groups: (i) the Control group without any intervention where the normal healing process would be monitored and compared, (ii) the Hydrogel group (where Hydrogel without the active substance would be applied once daily), and (iii) the Hydrogel-Porphyrin group (where the hydrogel with the encapsulated active substance of porphyrin would be applied once daily).

The Hydrogel group was deemed necessary in our study in order to investigate any effect that it might have (positive or negative). Half an hour after the initiation of anesthesia, dexmedetomidine was reversed with an intramuscular injection of atipamezole (1 mg/kg).

The substances were applied to the two rat groups on the day of surgery (day 0) and for 20 consecutive days. All rats were removed from their home cages, restrained, and inspected seven days a week, between 10-11 am. The Control rats were submitted only to restraint and inspection, in order for their manipulation and potential stress to be similar to the other groups. The Hydrogel and Hydrogel-Porphyrin groups were restrained, inspected, and the respective substances were applied to their skin defects from the pre-filled syringes.

Porphyrin synthesis

**Figure 2:**
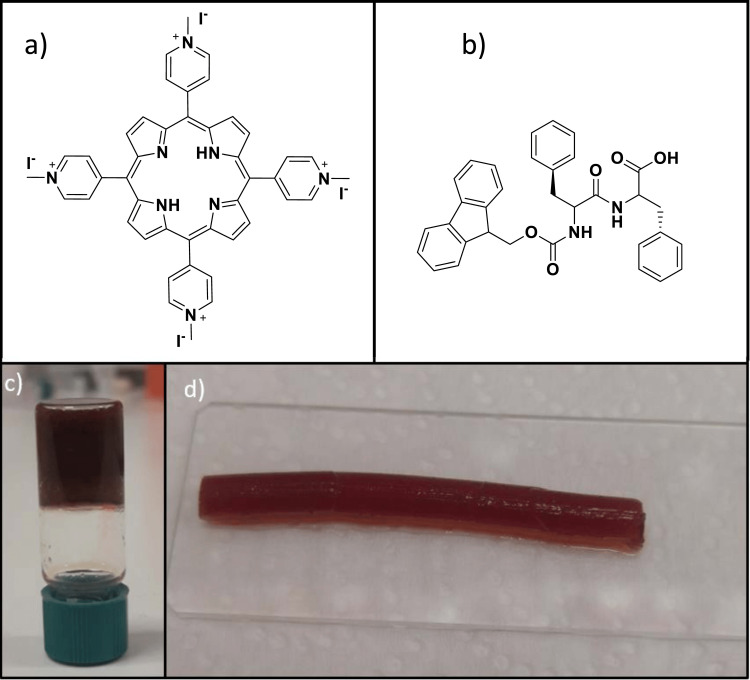
Chemical formulas and macroscopic images of the porphyrin encapsulated into the Fmoc-FF hydrogel. Chemical formulas of (a) (TMPyP^4+^)I_4_^-^ and (b) Fmoc-FF). Macroscopic images of the porphyrin encapsulated into the Fmoc-FF hydrogel, (c) and (d). Gel formed in a vial; the vial with a green cap is inverted in order to show the self-supporting nature of the hydrogel (c) and following syringe extrusion (d). (TMPyP^4+^)I_4_^- ^: porphyrin; Fmoc-FF: N-fluorenylmethoxy carbonyl- L-Phenylalanine-L-Phenylalanine

The water-soluble 5,10,15,20-tetra-N-methyl-4-pyridyl)porphyrin tetra iodide ([TMPyP^4+^]I_4_^-^) used for the hydrogel formation was prepared according to previously published work [21] and is illustrated in Figure 2a. Three concentrations of porphyrin were evaluated for encapsulation: 0.312 mM, 0.6 mM, and 1 mM. The two higher concentrations of porphyrin led to the formation of rigid hydrogels that were difficult to extrude and manipulate properly. Therefore, we opted for the lower concentration, since it allowed the formation of a strong hydrogel that could be at the same time smoothly extruded and applied to the skin.

Hydrogel formation

The hydrogel-forming peptide Fmoc-FF was purchased from Bachem Holding AG (Bubendorf, Switzerland) in the form of white lyophilized powder with purity greater than 95%. The chemical formula of the peptide is illustrated in Figure 2b. The fabrication of the hydrogel Fmoc-FF with the encapsulated porphyrin followed the solvent-switch method [11]. According to this method, the peptide powder is first dissolved into a “good solvent”, in this case, ethanol, and subsequently a “bad solvent”, in this case, water, which is added and triggers the self-organization process that leads to hydrogel formation. First, the peptide powder of the Fmoc-FF (5 mg) was dissolved in 0.25 ml pure ethanol using heating at 60^o ^C and sonication. Subsequently, the porphyrin ([TMPyP^4+^]I_4_^-^) was dissolved in 0.75 ml of sterilized water and it was transferred into the peptide solution triggering the self-organization process. The final porphyrin concentration was 0.312 mM and the molar ratio peptide to porphyrin was 30 to 1. The samples were placed at room temperature overnight, leading to the formation of stable hydrogels (Figures 2c, 2d). Two different morphologies of the hydrogel are illustrated depending on the formation vessel; i.e. inside a glass vial (Figure 2c), or extruded from a syringe (Figure 2d). The latter is an extremely useful property for biomedical applications since it allows hydrogel injection from a syringe onto a target tissue.

Evaluation of the healing process

Planimetry of The Defects

**Figure 3:**
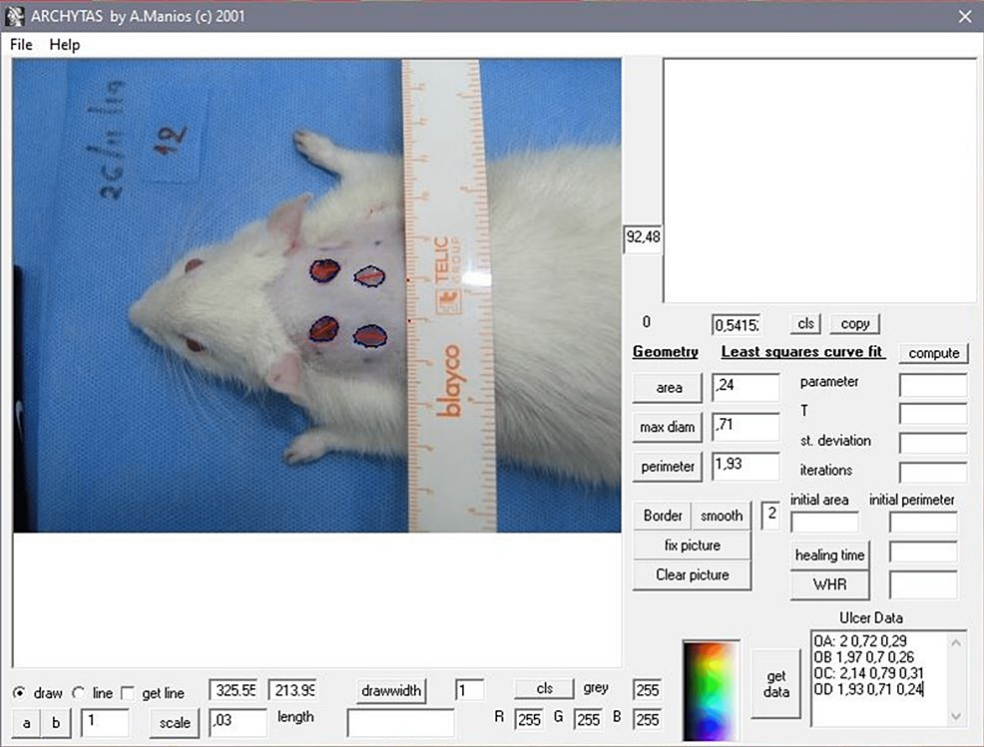
A screenshot from the Archytas program evaluating the baseline parameters of the four skin defects on the day of surgery (day 0).

Additionally to day 0 (baseline day of defect creation), at the third, seventh, 10th, and 20th postoperative days, the animals were anesthetized and the lesions were photographed by an Olympus digital camera (Model no. C-5060, 5.1 MegaPixel; Olympus Corporation, Tokyo, Japan). The animals and the camera were always positioned in the same orientation to the light and at the same distance of 15 cm. The assessment of the course of the defects was carried out by two independent researchers with the special planimetry program "Archytas" [20]. This program stores the wound border data during each photography session (Figure 3).

Depending on the size of the defect, which decreased over time due to the healing process, 6-12 points of evaluation were placed on the edges of the defects. At each session, a high-accuracy computation was conducted by the planimetry program of the healing process resulting in the defect surface parameters, which were the perimeter, the diameter, and the area of the defect. Next, the ratio of the final value of each parameter of each time point (third, seventh, 10th, and 20th postoperative days) per the initial baseline value of the deficit at the time of operation was estimated and compared, in order to monitor the healing process, expressed as a percentage and adjusted to baseline values, as: deficit A day 3/day 0, deficit B day 7/day 0, deficit C day 10/day 0, and deficit D day 20/day 0. Specifically, ratio index = (value i_th_ time/value baseline) x 100.

Pathology of The Defects

After the photographs, samples were collected with a 6 mm biopsy punch in a left-to-right orientation. The biopsy tissues were fixed in phosphate-buffered formaldehyde 10% solution for 24 hours, embedded in paraffin, and routinely stained with hematoxylin and eosin (H&E) staining. Sectioning was performed at 4 μm in Thermo Scientific™ HM 355S Automatic Microtome (Thermo Fisher Scientific Inc., Waltham, Massachusetts, United States). The samples of each rat of the third, seventh, 10th, and 20th postoperative days were evaluated blindly by two independent pathologists and graded numerically for the parameters of granulation, ulceration, and scar tissue formation as follows: 0=none, 1=mild, 2=moderate, and 3=severe. The aforementioned parameters were selected as indicators of wound healing based on their well-known role in tissue repair [22].

Immunohistochemical Evaluation

Immunohistochemistry was carried out by using standard procedures in all 192 tissue specimens. The sections were stained with antibodies against interleukin-6 (IL-6) (clone 1.2-2B11-2G10 (Abcam PLC, Cambridge, United Kingdom)/at dilution 1:200) and smooth muscle actin (SMA) (clone 1A4 (Abcam PLC)/at dilution 1:200). Antigen retrieval was performed at pH 6. The Dako EnVision visualization system was used (Agilent Technologies, Inc., Santa Clara, California, United States). DAB (3,3-diaminobenzidine) was used as a chromogen and hematoxylin as a counterstain. The immunohistochemical evaluation of IL-6 and SMA was based on the percentage of staining expression and on intensity. Both immunohistochemical stains aimed to help assess the healing process in the Control group, the Hydrogel group, and the Hydrogel-Porphyrin group.

IL-6 immunoexpression was evaluated in lymphocytes in order to assess the inflammatory stage of the tissue repair process. Staining percentage of lymphocytes was graded as 0=no staining, 1=1-10%, 2=11-50%, and 3=>50%, while intensity was graded as 0=no staining, 1=mild intensity, 2=moderate intensity, and 3=intense staining.

SMA was assessed in smooth muscle fibers in order to observe the remodeling stage, which represents the last stage of wound healing and leads to scar tissue formation, and was graded accordingly. More specifically, staining percentage was graded as 0=no staining, 1=1-10%, 2=11-50%, and 3=>50%, while staining intensity was evaluated as grade 0=no staining, 1=mild intensity, 2=moderate intensity, and 3=intense staining.

Evaluation of Topical and Systemic Safety of the Applications

In order to evaluate the topical safety of the applied gels, the lesions were evaluated daily during the gel application for the potential development of redness, inflammation, etc.

For the evaluation of the potential systemic impact of the applied gels, two blood collections were performed, one at baseline (before the creation of the skin defects) and one at the end of the experimental protocol (21st day) for the assessment of liver and kidney function. Blood collection was conducted using a restrainer (MLA5022 Rodent Restrainer). Local anesthetic cream (EMLA 5%, AstraZeneca PLC, Cambridge, United Kingdom) was applied on the rats’ tails half an hour prior to the puncture. Then each rat was placed within a restrainer, their tail was immersed in lukewarm water in order to achieve vasodilation, following which the lateral coccygeal vein was punctured with a safety winged IV needle. Blood was collected in a K3EDTA tube for a complete blood count (ADVIA® 2120i Hematology System, Siemens Healthineers, Erlangen, Germany) and in a plain test tube for biochemical analysis. The serum for biochemical analysis was separated after centrifugation at 3000 rpm for 10 minutes and stored in a deep freezer (-78° C). After centrifugation, the sera were inspected for the presence of hemolysis or lipaemia and were found free. The parameters evaluated from the complete blood count were: red blood cells (RBC), hemoglobin (HGB), hematocrit (HCT), white blood cells (WBC), neutrophils (NEUT), and platelets (PLTs), while the biochemical parameters evaluated were: urea (Ur), creatinine (Cr), total protein (TP), albumin (ALB), serum glutamic-pyruvic transaminase (SGPT), and alkaline phosphatase (ALP) (ADVIA 1200 Chemistry Analyzer, Siemens Healthineers).

Statistical analysis

Data were expressed as mean value ± standard deviation (SD) for continuous variables and as median (IQR) for histomorphometric variables. The Shapiro-Wilks test was used for normality analysis of the continuous variables. Comparison of the ratio index of the parameters and biochemical indices between groups was analyzed using the one-way ANOVA model. Pairwise comparisons were performed using the Bonferroni test. The Welch test and the Games-Howell tests were used in case of unequal variance between groups. Comparison of histomorphometric variables between groups was performed using the Kruskal-Wallis test and Dunn test adjusted by the Benjamini-Hochberg false discovery rate (FDR) method for pairwise comparisons. All tests were two-sided, and statistical significance was set at p<0.05. All analyses were performed using IBM SPSS Statistics for Windows, Version 21.0 (Released 2012; IBM Corp., Armonk, New York, United States).

## Results

Planimetry

Three parameters were evaluated with the planimetry method of Archytas as follows. As described previously, the ratio index of the perimeter, the diameter, and the area of each time point (third, seventh, 10th, and 20th postoperative day) per the initial baseline values at the time of operation were evaluated in order to monitor the healing process expressed as a percentage: Ratio index = (value i_th_ time/value baseline) x 100. The baseline measurements of the three parameters between the three groups did not have any statistically significant difference (data available upon request).

Young Animals

Regarding the ratio index of the perimeter of the defect, the analysis of the data of planimetry revealed a lower value in the Hydrogel-Porphyrin group for all the time points when compared to the Control group. These differences were statistically significant for the third, 10th, and 20th days. The same beneficial effect was evident when the comparison was performed between the Hydrogel-Porphyrin versus the Hydrogel group; in the latter comparison, statistically significant differences were evident on the 10th and 20th days (Table 1).

**Table 1:** Comparison of the ratio index of the perimeter of the skin defects of the three young rat groups on the four evaluation timepoints. All variables are presented as mean (%) ± standard deviation * p<0.05 vs Control, ** p<0.005 vs Control, † p<0.05 vs Hydrogel, †† p<0.005 vs Hydrogel

Ratio index day/baseline	Groups			p-value
	Control	Hydrogel	Hydrogel-Porphyrin	
Perimeter 3^rd^	87.03±11.01	72.73±3.72 **	71.06±5.88 **	<0.005
Perimeter 7^th^	42.49±5.91	41.07±5.94	35.63±8.52	0.166
Perimeter 10^th^	34.41±10.81	32.57±7.88	18.68±2.95 ** ††	<0.005
Perimeter 20^th^	34.86±7.43	37.11±10.71	17.55±4.61** ††	<0.005

Regarding the ratio index of the diameter of the defect, the analysis of the data of planimetry revealed a statistically significant beneficial effect in the Hydrogel-Porphyrin group on the third, 10th, and 20th days with lower values when compared to the Control group, and on the 10th and 20th days when compared to the Hydrogel group (Table 2).

**Table 2:** Comparison of the ratio index of the diameter of the skin defects of the three young rat groups on the four evaluation timepoints. All variables are presented as mean (%) ± standard deviation * p<0.05 vs Control, ** p<0.005 vs Control, ^†^ p<0.05 vs Hydrogel, ^††^ p<0.005 vs Hydrogel

Ratio index day/baseline	Groups			p-value
	Control	Hydrogel	Hydrogel-Porphyrin	
Diameter 3^rd^	93.47±13.63	77.32±4.56 **	72.07±6.48 **	<0.005
Diameter 7^th^	44.96±8.16	41.20±6.27	36.58±6.98	0.112
Diameter 10^th^	37.04±9.98	36.43±10.67	19.17±4.26 ** ††	<0.005
Diameter 20^th^	40.64±11.07	41.52±12.69	19.49±6.56 ** ††	<0.005

Regarding the ratio index of the area of the defect, the analysis of the data of planimetry revealed that the Hydrogel-Porphyrin group had a lower area value for all the time points indicating a favorable healing progression when compared to the other two groups. Statistical significant differences were observed on the third, 10th, and 20th days when compared to the Control group, while when compared to the Hydrogel group, this difference was significant only on the 10th day (Table 3).

**Table 3:** Comparison of the ratio index of the area of the skin defects of the three young rat groups on the four evaluation timepoints. All variables are presented as mean (%) ± standard deviation * p<0.05 vs Control, ** p<0.005 vs Control, ^†^ p<0.05 vs Hydrogel, ^††^ p<0.005 vs Hydrogel

Ratio index day/baseline	Groups			p-value
	Control	Hydrogel	Hydrogel-Porphyrin	
Area 3^rd^	69.62±20.39	45.92±7.39 **	45.15±9.54 **	<0.005
Area 7^th^	16.04±4.62	14.15±4.11	11.99±7.06	0.406
Area 10^th^	7.73±0.69	8.94±3.41	3.44±0.86 ** ††	<0.005
Area 20^th^	10.34±3.21	6.99±5.86	2.03±2.19 ** †	<0.005

Mature Animals

A similar pattern was observed in the mature animals. Regarding the ratio index of the perimeter of the defect, the Hydrogel-porphyrin group had a statistically significantly lower ratio when compared to the Control and Hydrogel groups for all the time points evaluated (Table 4).

**Table 4:** Comparison of the ratio index of the perimeter of the skin defects of the three mature rat groups on the four evaluation timepoints. All variables are presented as mean (%) ± standard deviation * p<0.05 vs Control, ** p<0.005 vs Control, ^†^ p<0.05 vs Hydrogel, ^††^ p<0.005 vs Hydrogel

Ratio index day/baseline	Groups			p-value
	Control	Hydrogel	Hydrogel-Porphyrin	
Perimeter 3^rd^	87.97±6.00	85.83±4.80	75.99±10.65 * †	0.017
Perimeter 7^th^	49.38±8.83	45.92±7.69	35.87±6.12 * †	0.008
Perimeter 10^th^	35.21±6.92	34.99±8.97	20.71±3.90 ** ††	<0.005
Perimeter 20^th^	44.29±3.34	40.85±9.40	23.62±4.91 ** ††	<0.005

Regarding the ratio index of the diameter of the defect, the Hydrogel-Porphyrin group had a statistically significant lower value when compared to both the Control and the Hydrogel groups on the seventh, 10th, and 20th day (Table 5).

**Table 5:** Comparison of the ratio index of the diameter of the skin defects of the three mature rat groups on the four evaluation timepoints. All variables are presented as Mean (%) ± standard deviation * p<0.05 vs Control, ** p<0.005 vs Control, ^†^ p<0.05 vs Hydrogel, ^††^ p<0.005 vs Hydrogel

Ratio index day/baseline	Groups			p-value
	Control	Hydrogel	Hydrogel-Porphyrin	
Diameter 3^rd^	88.25±6.88	82.51±10.99	78.72±11.35	0.250
Diameter 7^th^	51.97±6.57	45.44±7.05	35.40±2.47 ** †	<0.005
Diameter 10^th^	35.84±7.36	37.88±11.79	22.44±5.14 * †	<0.005
Diameter 20^th^	48.77±9.44	44.93±11.01	28.85±7.51 ** ††	<0.005

Regarding the ratio index of the area of the defect, the analysis of the data of planimetry revealed that the Hydrogel-Porphyrin group had a smaller area value for all the time points when compared to the other two groups. Statistical significant differences were observed for all the time points when compared to the Control group, while when compared to the Hydrogel group, this difference was significant only on the seventh, 10th, and 20th day (Table 6).

**Table 6:** Comparison of the ratio index of the area of the skin defects of the three mature rat groups on the four evaluation timepoints. All variables are presented as mean (%) ± standard deviation * p<0.05 vs Control, ** p<0.005 vs Control, ^†^ p<0.05 vs Hydrogel, ^††^ p<0.005 vs Hydrogel

Ratio index day/baseline	Groups			p-value
	Control	Hydrogel	Hydrogel-Porphyrin	
Area 3^rd^	75.72±11.21	69.16±10.30	56.52±15.95 *	0.033
Area 7^th^	21.63±6.66	19.31±6.93	9.44±2.46 ** ††	<0.005
Area 10^th^	10.60±3.00	10.31±5.05	4.26±1.61 ** ††	<0.005
Area 20^th^	11.47±1.61	12.23±4.45	4.25±1.73 ** ††	<0.005

**Figure 4:**
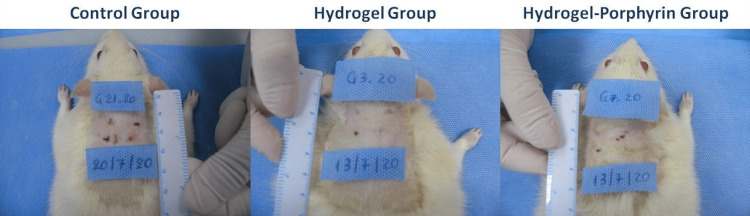
Skin defects of indicative animals from the three groups of the mature rats (Control, Hydrogel, and Hydrogel-Porphyrin) are shown macroscopically on the 20th postoperative day.

In Figure 4, the skin defects conducted on indicative animals from the three groups of the mature rats and their healing process on the 20th postoperative day are shown macroscopically. From their macroscopic monitoring during the experimental period regarding the evaluation of a potential local negative effect of Hydrogel and Hydrogel-Porphyrin application on the skin defects, the presence of infection or erythema was not observed.

Comparison of young and mature animals

In Table 7, the ratios of the three skin defect parameters of the young and mature rats of each experimental group (Control, Hydrogel, and Hydrogel-Porphyrin) are compared.

**Table 7:** Comparison of the ratio index of the three skin defect parameters evaluated (perimeter, diameter, and area) on the respective days between young and mature rats. A tendency for an increased healing rate in the young rats is noted, when compared to the mature rats. All variables are presented as mean (%) ± standard deviation; p-values with statistical significance are in bold.

Ratio index day/baseline	Control			Hydrogel			Hydrogel-Porphyrin		
	Young (n=8)	Mature (n=8)	p-value	Young (n=8)	Mature (n=8)	p-value	Young (n=8)	Mature (n=8)	p-value
Perimeter 3^rd^	87.03±11.01	87.97±6.00	0.857	72.73±3.72	85.83±4.80	<0.001	71.06±5.88	75.99±10.65	0.270
Perimeter 7^th^	42.49±5.91	49.38±8.83	0.143	41.07±5.94	45.92±7.69	0.180	35.63±8.52	35.87±6.12	0.948
Perimeter 10^th^	34.41±10.81	35.21±6.92	0.881	32.57±7.88	34.99 ±8.97	0.576	18.68±2.95	20.71±3.90	0.260
Perimeter 20^th^	34.86±7.43	44.29±3.34	0.018	37.11±10.71	40.85 ±9.40	0.470	17.55±4.61	23.62±4.91	0.023
Diameter 3^rd^	93.47±13.63	88.25±6.88	0.422	77.32±4.56	82.51 ±10.99	0.237	72.07±6.48	78.72±11.35	0.172
Diameter 7^th^	44.96±8.16	51.97±6.57	0.132	41.20±6.27	45.44 ±7.05	0.224	36.58±6.98	35.40±2.47	0.658
Diameter 10^th^	37.04±9.98	35.84±7.36	0.818	36.43±10.67	37.88 ±11.79	0.800	19.17±4.26	22.44±5.14	0.187
Diameter 20^th^	40.64±11.07	48.77±9.44	0.201	41.52±12.69	44.93 ±11.01	0.576	19.49±6.56	28.85±7.51	0.019
Area 3^rd^	69.62±20.39	75.72±11.21	0.536	45.92±7.39	69.16 ±10.30	<0.001	45.15±9.54	56.52±15.95	0.106
Area 7^th^	16.04±4.62	21.63±6.66	0.122	14.15±4.11	19.31 ±6.93	0.092	11.99±7.06	9.44±2.46	0.353
Area 10^th^	7.73±0.69	10.60±3.00	0.067	8.94±3.41	10.31 ±5.05	0.535	3.44±0.86	4.26±1.61	0.227
Area 20^th^	10.34±3.21	11.47±1.61	0.857	6.99±5.86	12.23 ±4.45	0.064	2.03±2.19	4.25±1.73	0.041

Histology and immunohistochemistry

**Figure 5:**
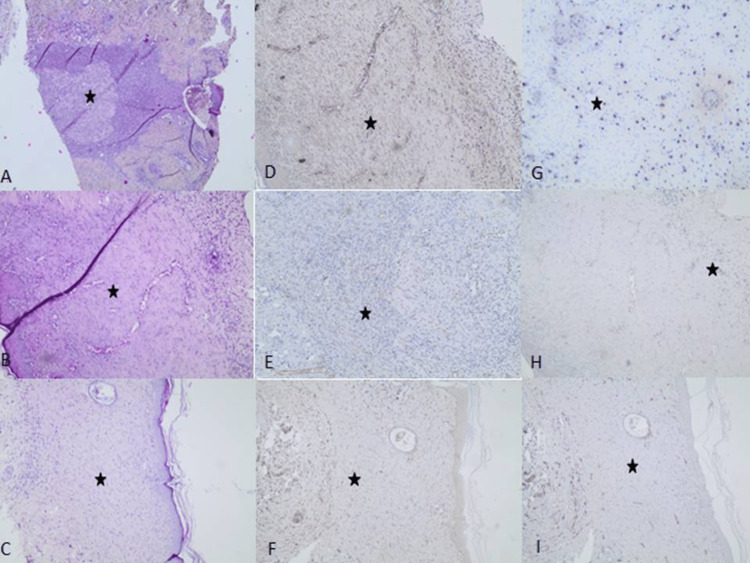
Indicative sections of mature rats. (A) H&E section of the Control group on the 20th day displaying increased granulation tissue with hypertrophic scar formation (asterisk highlighting the area of granulation tissue) (x100), (B) H&E section of the Hydrogel group on the 20th day displaying moderate granulation tissue and only mild hypertrophic scar tissue formation with a small focus of neutrophilic infiltration of the epidermis (asterisk highlighting the area of granulation tissue) (x100), (C) H&E section of the Hydrogel-Porphyrin group on the 20th day showing minimal granulation and normal scar tissue formation (asterisk showing the area of granulation tissue formation) (x100), (D) SMA staining (Control group) highlighting the smooth muscle fibers that contribute to the hypertrophic scar tissue formation (asterisk showing the positive staining of SMA in smooth muscle fibers) (score 2), (E) SMA staining (Hydrogel group) less than 10% (score 1) of the smooth muscle fibers (asterisk showing the moderate staining of SMA in smooth muscle fibers), (F) SMA stain (Hydrogel-Porphyrin group) being completely negative (score 0) (asterisk showing the absence of SMA stain in smooth muscle fibers), (G) Sparse IL-6 stain (Control group) in lymphocytes due to the increased amount of neutrophils (asterisk showing the IL-6 positive inflammatory cells) (score 2), (H) Focal IL-6 staining (asterisk showing focal IL-6 positive inflammatory cells) (score 1) in the Hydrogel group, I) IL-6 being completely negative (score 0) in the Hydrogel-Porphyrin group (asterisk showing the absence of IL-6 staining). H&E: Hematoxylin/Eosin, SMA: Smooth Muscle Actin, IL-6: Interleukin-6

Indicative histological findings for granulation, ulceration, and scar tissue formation of the three mature rat groups on day 20 on H&E sections, as well as on the immunohistochemistry parameters of IL-6 and SMA, in order to assess the quality of scar tissue formation, are shown in Figure 5.

The statistically significant different parameters of immunohistochemistry and histology are presented in Table 8 between the three treatment groups of young and mature rats.

**Table 8:** Immunohistochemistry and histology parameters with statistically significant differences between the three treatment groups of young and mature rats. All variables are presented as median (IQR); * p<0.05 vs Control, ^†^ p<0.05 vs Hydrogel IQR: Interquartile range, SMA: Smooth Muscle Actin

Rat Group	Time (post-operative day)	Variables	Groups			p-value
			Control	Hydrogel	Hydrogel-Porphyrin	
Young	3^rd^	SMA intensity	0.0 (1.0)	1.0 (1.0) *	1.0 (1.0) *	0.009
	7^th^	Scar Tissue formation	0.5 (2.3)	1.0 (0.5)	0.0 (0.5) †	0.027
	7^th^	Granulation	1.5 (1.0)	2.5 (1.0) *	2.0 (0.5)	0.019
	10^th^	Granulation	1.0 (0.5)	2.0 (2.0) *	1.0 (1.0)	0.017
Mature	3^rd^	Scar Tissue formation	1.0 (0.5)	2.5 (1.8) *	1.0 (0.1)	0.045
	3^rd^	Granulation	3.0 (0.3)	2.0 (1.8) *	3.0 (0.8) †	0.027

Evaluation of the systemic safety of the applications

Complete blood count and biochemical analysis of selected parameters of the young rats' blood samples taken before (Initial value) and after (End value) the creation of the skin defects and application of the substances are provided in Table 9. The statistical significance and p values of the comparisons between groups and within groups (Initial/End values) are indicated in Table 9.

**Table 9:** Comparison of complete blood count and biochemical parameters of the three treatment groups of the young animals. RBC: Red Blood Cells, HGB: Hemoglobin, HCT: Hematocrit, WBC: White Blood Cells, NEUT: Neutrophils, PLTs: Platelets, Ur: Urea, Cr: Creatinine, TP: Total Protein, ALB: Albumin, SGPT: Serum Glutamic-Pyruvic Transaminase, ALP: Alkaline Phosphatase ^a^p<0.05 vs Group Hydrogel, ^b^p<0.005 vs Group Hydrogel, ^c^p<0.05 vs Group Hydrogel-Porphyrin, ^d^p<0.005 vs Hydrogel-Porphyrin, ^e^p<0.05 vs beginning, ^f^p<0.005 vs beginning

Parameter	Young animal Groups	Initial value	End value
		Mean±SD	Mean±SD
RBC x10^6^/μL	Control	9.78±0.25^ b^	9.73±0.24
	Hydrogel	8.82±0.60	9.62±0.19^f^
	Hydrogel-Porphyrin	9.50±0.22^ a^	9.77±0.13
HGB gr/dL	Control	15.90±0.18	15.48±0.50^ e^
	Hydrogel	15.84±0.52	15.77±0.35
	Hydrogel-Porphyrin	15.66±0.38	15.58±0.44
HCT %	Control	58.25±2.22	54.13±1.90^ e^
	Hydrogel	54.33±3.68	56.18±1.50
	Hydrogel-Porphyrin	56.05±2.12	55.34±1.35
WBC x10^3^/μL	Control	7.04±2.27	6.41±1.23
	Hydrogel	6.72±1.12	6.55±0.56
	Hydrogel-Porphyrin	7.56±1.84	7.01±1.30
NEUT x10^3^/μL	Control	0.91±0.20	0.81±0.10
	Hydrogel	1.06±0.13	0.94±0.21
	Hydrogel-Porphyrin	1.09±0.11	0.95±0.35
PLTs x10^3^/μL	Control	601.00±88.10	638.67±71.61
	Hydrogel	503.46±98.05	575.00±64.50 ^e^
	Hydrogel-Porphyrin	573.96±62.92	638.62±27.88 ^e^
Ur mg/dL	Control	48.71±6.79	48.50±2.78
	Hydrogel	55.95±13.39	40.02±9.75^f^
	Hydrogel-Porphyrin	56.02±7.38	54.46±6.86^ b^
Cr mg/dL	Control	0.202±0.08	0.260±0.03^ c^
	Hydrogel	0.251±0.05	0.249±0.06
	Hydrogel-Porphyrin	0.254±0.04	0.324±0.05 ^e a^
TP g/dL	Control	7.23±0.12	7.43±0.16^ a^
	Hydrogel	7.18±0.18	7.10±0.16
	Hydrogel-Porphyrin	7.03±0.19	7.20±0.19
ALB g/dL	Control	4.68±0.21	4.48±0.13
	Hydrogel	4.56±0.18	4.35±0.18
	Hydrogel-Porphyrin	4.50±0.27	4.44±0.21
SGPT IU/L	Control	103.55±21.91	93.40±11.04
	Hydrogel	108.82±42.06	110.49±28.01
	Hydrogel-Porphyrin	81.04±17.52	74.29±16.75^ a^
ALP IU/L	Control	45.17±24.98	35.17±18.51
	Hydrogel	63.25±23.89	32.16±11.52
	Hydrogel-Porphyrin	50.38±19.12	48.38±19.94

Complete blood count and biochemical analysis of selected parameters of the mature rats' blood samples taken before (Initial value) and after (End value) the creation of the skin defects and application of the substances are provided in Table 10. The statistical significance and p values of the comparisons between groups and within groups (Initial/End values) are indicated in Table 10.

**Table 10:** Comparison of complete blood count and biochemical parameters of the three treatment groups of the mature animals. RBC: Red Blood Cells, HGB: Hemoglobin, HCT: Hematocrit, WBC: White Blood Cells, NEUT: Neutrophils, PLTs: Platelets, Ur: Urea, Cr: Creatinine, TP: Total Protein, ALB: Albumin, SGPT: Serum Glutamic-Pyruvic Transaminase, ALP: Alkaline Phosphatase ^a^p<0.05 vs Group Hydrogel, ^b^p<0.005 vs Group Hydrogel, ^c^p<0.05 vs Group Hydrogel-Porphyrin, ^d^p<0.005 vs Hydrogel-Porphyrin, ^e^p<0.05 vs beginning, ^f^p<0.005 vs beginning

Parameter	Mature animal Groups	Initial value	End value
		Mean±SD	Mean±SD
RBC x10^6^/μL	Control	9.82±0.38^ c^	9.40±0.17^f^
	Hydrogel	9.96±0.30	9.26±0.29^f^
	Hydrogel-Porphyrin	9.36±0.26^ b^	9.24±0.22
HGB gr/dL	Control	16.07±0.67	16.00±0.51
	Hydrogel	16.24±0.64	15.63±0.32^f^
	Hydrogel-Porphyrin	15.60±0.54	15.90±0.45
HCT %	Control	60.45±2.71	58.35±1.55^ e ^
	Hydrogel	61.25±2.38	56.76±1.78^f^
	Hydrogel-Porphyrin	57.80±2.54^ a^	58.04±2.05
WBC x10^3^/μL	Control	4.39±0.45	4.13±0.48
	Hydrogel	4.25±0.61	4.22±0.55
	Hydrogel-Porphyrin	4.25±0.61	4.22±0.55
NEUT x10^3^/μL	Control	0.70±0.24	0,81±0.29^ e^
	Hydrogel	0.75±0.19	0.92±0.14^e^
	Hydrogel-Porphyrin	0.94±0.27	1.04±0.20^ e^
PLTs x10^3^/μL	Control	501.83±56.17	468.50±77.98
	Hydrogel	442.75±78.82	427.00±93.75
	Hydrogel-Porphyrin	483.13±74.96	471.25±45.56
Ur mg/dL	Control	44.59±3.47	46.32±8.10
	Hydrogel	41.17±5.11	40.83±5.72
	Hydrogel-Porphyrin	41.49±4.83	45.77±6.62
Cr mg/dL	Control	0.462±0.11	0.505±0.04
	Hydrogel	0.443±0.06	0.469±0.03
	Hydrogel-Porphyrin	0.424±0.11	0.486±0.03
TP g/dL	Control	7.35±0.31^c^	7.38±0.26^ a^
	Hydrogel	7.15±0.19	7.11±0.15
	Hydrogel-Porphyrin	6.94±0.24	7.19±0.21^e^
ALB g/dL	Control	4.83±0.47	4.47±0.19^ e^
	Hydrogel	4.63±0.22	4.29±0.20 ^e^
	Hydrogel-Porphyrin	4.57±0.46	4.39±0.19
SGPT IU/L	Control	102.30±8.4^b^	74.38±10.33^ f^
	Hydrogel	103.96±12.18	75.06±14.93
	Hydrogel-Porphyrin	81.61±18.73^b^	73.09±17.43^ f^
ALP IU/L	Control	40.00±22.95	144.17±55.71^ f ^
	Hydrogel	63.00±29.49	115.00±42.34^ f^
	Hydrogel-Porphyrin	50.00±23.49	113.63±39.61^f^

## Discussion

Hydrogels are widely known components of wound dressings in clinical practice. They provide a moist environment which is advantageous for wound healing while also absorbing the wound’s exudates [7,23]. Hydrogels are currently used mainly as vehicles that are combined with other active molecules meant to accelerate wound healing. Such a combination was tested in our study, namely Hydrogel-Porphyrin as a multifunctional wound healing gel. The porphyrin ([TMPyP^4+^]I_4_^-^) utilized in this work, has been functionalized with methyl-pyridyl groups (Figure 2a) in order to achieve the solubility of the photosensitizer in aqueous media, which is essential for the formation of the hydrogel.

A simple, accurate, and objective method of wound healing assessment through measurements of ulcer dimensions and calculation of wound margin advancement is introduced in this study [20]. The accuracy of the program is high (over 95%) as was repeatedly tested by measurement of known surfaces. A similar evaluation method has been used in mice skin defects [24].

In the young rats, in all three parameters measured (perimeter, diameter, and area), there was a statistically significant difference (p<0.005) in favor of the Hydrogel-Porphyrin group on the 10th and 20th postoperative days when compared to Hydrogel alone and for the third, 10th, and 20th day when compared to the Control. In the Hydrogel group, there appears to be a beneficial third-day effect compared to the Control group on all three parameters. It could be assumed that this may be due to the early hydration of the skin defect, which is a known property of hydrogels [7].

In the mature rats, a significant statistical beneficial effect was recorded for the Hydrogel-Porphyrin group for the perimeter on the third and seventh day (p<0.05) and on the 10th and 20th day (p<0.005) when compared to Hydrogel and Control groups. For the diameter and area, this effect was evident on the seventh, 10th, and 20th day (p<0.005) when the Hydrogel-Porphyrin group was compared to the Hydrogel and Control groups. Although the percentage of healing was superior in the young animals in all groups and in all time points the beneficial effect of the Hydrogel-Porphyrin application was more conspicuous in the mature animals, both regarding the level of statistical significance and time.

The comparison of the two age groups showed that in both groups, all the parameters evaluated followed a similar healing pattern. There was a tendency for an increased healing rate in the young rats when compared to the mature rats. In the Control groups, on the defects of which no substance was applied, the healing process as regards the parameter of the 20th day perimeter was statistically significantly decreased compared to the mature rats. The same tendency was observed for the Hydrogel group, with a statistically significant difference in the parameters, perimeter, and area on the third day, while for the Hydrogel-Porphyrin group, statistically significant differences were observed in all parameters on the 20th day. The delayed healing rate in the mature animals, in agreement with Gosain and DiPietro [25], may be the reason why, in this age group, the statistically significant differences in the beneficial effect of Hydrogel-Porphyrin compared to Hydrogel alone were evident at earlier time points, in comparison to the young animals.

Complete blood count and biochemical parameters were investigated as indicators of inflammation and toxicity. From the complete blood count of the young animals, the majority of the parameters selected for statistical evaluation neither revealed statistically significant differences either between groups or between the initial and end values, nor exceeded reference values [26]. Although animals were randomly allocated for each group, RBC values at baseline were statistically significantly lower in the Hydrogel group when compared to Control and Hydrogel-Porphyrin groups. Comparison of RBC values at the end of the study revealed no statistically significant difference between the three groups, but the Hydrogel group was higher when compared with its initial value. Furthermore, RBC and HCT were the only parameters higher than the reference values both in initial and end measurements. This may be attributed to fear or excitement and a consequent splenic contraction [27]. The same pattern was recorded for the mature animals. The evaluation of the biochemical parameters of both young and mature animals revealed that the application of Hydrogel or Hydrogel-Porphyrin had either no effect, or on the parameters where statistically significant differences were noted between time points and treatment groups, the values were between reference ranges [26].

The wound healing repair process occurs in four stages, namely hemostasis, inflammatory stage, proliferative stage, and remodelling stage. Each stage contributes to the formation of healthy granulation tissue and normotrophic scar tissue formation. Histologically, granulation tissue is composed of sprouting capillaries that tend to protrude from the surface of a healing wound producing minute red granules. The new capillaries at first are solid but shortly develop lumens containing blood cells (angiogenesis); some of these persist while others are resorbed. Cells and proliferating capillaries are the two major components of granulation tissue. The cells are chiefly fibroblasts and inflammatory cells, macrophages, lymphocytes, plasma cells, and neutrophils depending on the stage and development of the granulation tissue and the presence of infection. Fibroblasts appear early and form collagen. As the tissue matures, the fibroblasts look less active and eventually become the main cells of the final scar. Similarly, the vascular tissue is reabsorbed and in the final scar may be inconspicuous. However, errors during this phase can lead to excessive wound healing with the formation of hypertrophic scar or keloid [22,28].

In our study, histological evaluation of the wound healing process demonstrated that both young and mature animals treated with the Hydrogel-Porphyrin combination displayed the least hypertrophic scar tissue formation when compared with the group of animals treated with Hydrogel alone. Furthermore, granulation tissue was also less prominent on the 7th and 10th day in the young animals treated with Hydrogel-Porphyrin, compared to those treated with Hydrogel, although not significantly. This is probably explained by the fact that chronic wounds result in persistent granulation tissue, therefore this finding may also suggest that Hydrogel-Porphyrin accelerates wound healing.

This study may be considered to have a translational limitation because of the anatomical difference of the rat model compared to humans, which was described in the Introduction. The presence of the muscle *panniculus carnosus *on the rats’ dorsal area may contribute to increase their wound healing rate, compared to humans. However, this area is considered the safest for the creation of skin defects without having the risk of their causing further trauma to the wounds. Additionally, it would have been interesting to evaluate a larger concentration of the encapsulated Porphyrin. This however, would need a twofold increase of the number of animals used, which would not be in line with the principle of Reduction of the 3 Rs (Replacement, Reduction, Refinement) [29,30]. Furthermore, this study investigated Porphyrin’s properties on the healing of skin defects for the first time, for which its dose-related action was not the primary endpoint and may well be a further investigation. The current concentration was selected based on its properties described previously, allowing smooth application on the skin.

## Conclusions

The application of porphyrin encapsulated into hydrogel upon the skin defects benefited the healing process in both young and mature animal groups, as evidenced by the statistically significant findings of planimetry. The beneficial healing process was more pronounced in the mature animals both in the level of statistical significance as well as regarding time (evident already on the third day of healing), possibly because of porphyrin assisting the reduced normal rate of healing, which is observed in mature organisms. The above-mentioned approach offers an innovative direction for the use of peptide-porphyrin hydrogels in various applications such as drug delivery and tissue regeneration following trauma. Additional studies may take our findings further by evaluating delivery methods of porphyrin encapsulated into hydrogel, which combines accurate dosage, cohesion, and ease of application to skin defects that could be invaluable for their treatment in clinical practice.
